# Granulomatosis With Polyangiitis Presenting as Pulmonary Nodules: A Case Study and Literature Review

**DOI:** 10.1155/crii/6977376

**Published:** 2026-07-27

**Authors:** Yanghang Chen, Haihong Zheng, Liuyang Gong

**Affiliations:** ^1^ Department of Respiratory and Critical Care Medicine, Zhejiang Taizhou Hospital, Linhai, China; ^2^ Department of Pathology, Zhejiang Taizhou Hospital, Linhai, China

**Keywords:** case study, granulomatosis with polyangiitis, myeloperoxidase-antineutrophil cytoplasmic antibody (MPO-ANCA), pulmonary nodules

## Abstract

This article presents a case of myeloperoxidase‐antineutrophil cytoplasmic antibody (MPO‐ANCA)–positive granulomatosis with polyangiitis (GPA) presenting initially as chronic cough. Pulmonary shadows persisted and progressed despite antibiotic therapy. The diagnosis was confirmed by lung biopsy showing granulomatous inflammation with vasculitis, and the patient improved significantly with glucocorticoids combined with cyclophosphamide. This report, supplemented by a review of relevant literature, aims to enhance clinical awareness of this disease and emphasizes the importance of considering vasculitis and obtaining pathological confirmation in cases of refractory pulmonary lesions.

## 1. Clinical Data

The patient was a 67‐year‐old male with no significant past medical history. In May 2025, he developed a paroxysmal cough productive of scant white sputum, which showed no correlation with body position or diurnal rhythm. His symptoms persisted despite symptomatic treatment at a local hospital, prompting his visit to our institution. A chest CT scan performed on June 6, 2025, revealed scattered inflammatory and fibroproliferative opacities in both lungs, mild bronchiectasis in the upper lobes and right middle lobe, and multiple small nodules (partially calcified) in both lungs. Laboratory tests including complete blood count and C‐reactive protein were within normal limits. Subsequent empirical therapy with cefdinir and moxifloxacin yielded no clinical improvement. Follow‐up CT on July 17, 2025, showed progressive infectious consolidations in the right middle/lower lobes and left upper lobe, with an increased number of bilateral small nodules (partially calcified) (Figure 1). The patient was subsequently hospitalized for further management.

**Figure 1:**
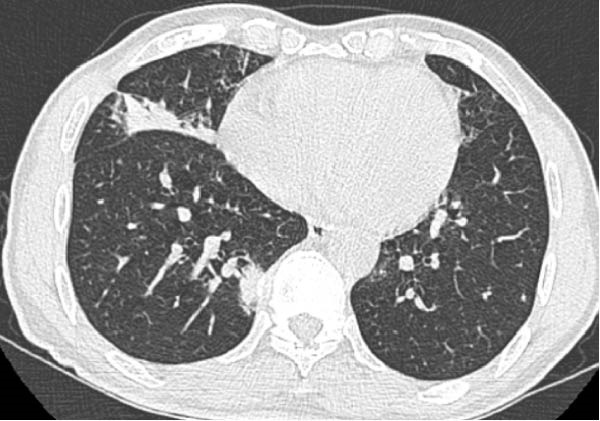
CT scan revealed patchy opacities with ill‐defined margins in the right middle lobe and right lower lobe of the patient’s lung.

Following admission, comprehensive laboratory investigations revealed an elevated myeloperoxidase‐antineutrophil cytoplasmic antibody (MPO‐ANCA) level of 49.7 AU/mL. Other laboratory results are shown in Table 1. Endobronchial ultrasound–guided biopsy and bronchoalveolar lavage were performed. Histopathological examination demonstrated minimal lung tissue with focal lymphocytic infiltration, while metagenomic next‐generation sequencing of bronchoalveolar lavage fluid detected no pathogenic microorganisms.

**Table 1:** Laboratory findings of the reported case.

Category	Test item	Result	Clinical significance
Immune‐mediated markers	MPO‐ANCA	49.7 AU/mL	Suggests vasculitis
	PR3‐ANCA	Negative	—
	Antinuclear antibody (ANA)	Normal	No evidence of other CTD
	ESR/CRP	Normal	Early or localized stage
Infectious workup	Interferon‐gamma release assay (IGRA/T‐SPOT.TB)	Negative	Negative for tuberculosis
	(1,3)‐β‐D‐glucan/galactomannan	Negative	No evidence of active fungal infection
	Sputum bacterial and fungal cultures	Negative	No bacterial/fungal growth
	mNGS (BALF)	Negative	No evidence of rare pathogens
Tumor markers	CEA, NSE, SCC	Normal	Low probability of malignancy
	AFP, CA19‐9, CA125	Normal	No evidence of common carcinomas
Organ involvement	Nasal endoscopy	Sinusitis	Upper respiratory involvement
	Audiometric testing	Mixed hearing loss	Otorhinolaryngological involvement

The poor response to broad‐spectrum antibiotic therapy and the paradoxical radiological progression provided the first clinical clue that the pulmonary lesions were noninfectious in nature. Given the persistent suspicion of autoimmune diseases such as vasculitis, methylprednisolone 20 mg daily was initiated on July 23, 2025. The patient subsequently showed symptomatic improvement in cough.

A follow‐up CT scan on August 6, 2025, revealed overall resolution of pulmonary patches and nodular opacities, with a partial reduction in the size of multiple nodules (Figure 2). This radiographic improvement supported the efficacy of corticosteroid therapy, and continued prednisone treatment was recommended. However, the patient self‐discontinued the medication.

**Figure 2:**
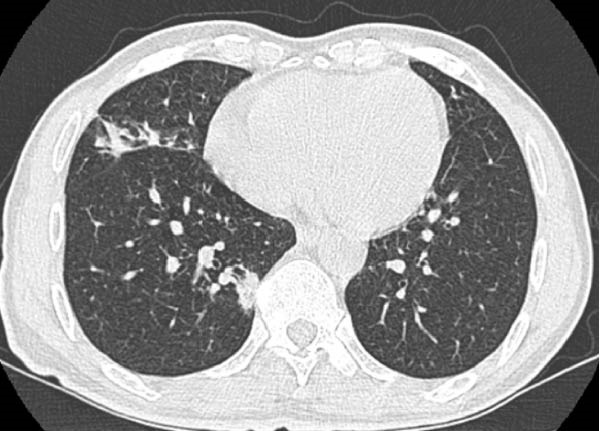
Overall absorption of the patchy and nodular opacities in both lungs is noted compared to prior studies. Some of the multiple nodules appear to have possibly decreased in size.

He presented again on September 24, 2025, due to a worsened cough. A repeat contrast‐enhanced CT was obtained, which showed progressive pulmonary disease. The imaging demonstrated an increase in the number and size of patchy opacities, which had ill‐defined margins (Figure 3). Notably, a dominant lesion in the right lower lobe had grown to 40 × 18 mm with moderate homogeneous enhancement. Given these findings, he was readmitted for a CT‐guided lung biopsy.

**Figure 3:**
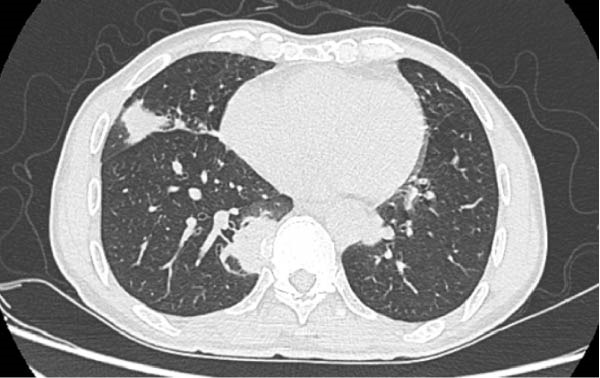
The multiple patchy opacities in both lungs remain largely stable compared to the previous study. The largest lesion is currently located in the right lower lobe, measuring ~40 × 18 mm.

Histopathological examination revealed acute and chronic mucosal inflammation with focal granulomatous changes and alveolar epithelial hyperplasia. Further analysis showed alveolar edema, architectural destruction, multifocal neutrophilic infiltration in the interstitium, obliterated small vessel structures, scattered multinucleated giant cells, and lymphocytic infiltration (Figure 4). Special staining results are silver methenamine (−), PAS (−), SMA (partial‐), CD34 (partial‐), argentaffin (+), GMS (−), acid‐fast (−). Elastic fiber staining (EVG/special stain) demonstrated focal fragmentation and loss of the internal elastic lamina, providing direct histopathological evidence of necrotizing vasculitis (Figure 5). The composite histopathological features confirmed the diagnosis of pulmonary granulomatosis with polyangiitis (GPA).

**Figure 4:**
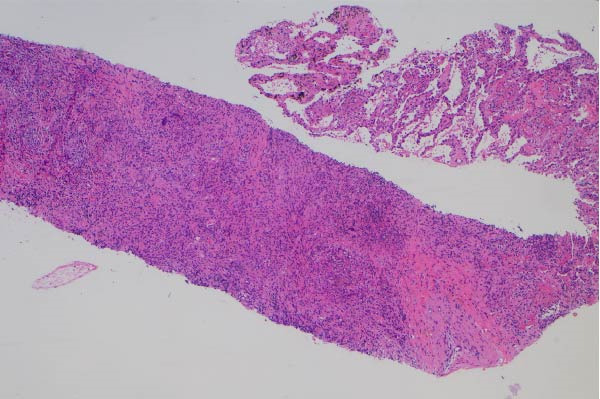
HE × 100: Fibroproliferative changes of the pulmonary interstitium, accompanied by a histiocytic reaction, scattered multinucleated giant cells, and infiltration of lymphocytes and neutrophils.

**Figure 5:**
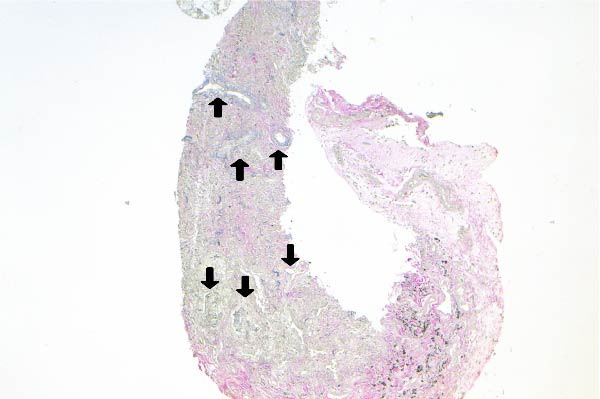
The upper image shows the result of elastic fiber staining (EVG/special stain). The upward arrow indicates relatively intact elastic fibers within the vessel wall, whereas, in contrast, the downward arrow points to areas of the vessel wall where staining is absent, suggesting a loss of the corresponding elastic fibers in these vascular walls.

The patient began a treatment regimen of daily cyclophosphamide (0.1 g) and morning methylprednisolone (20 mg). At the 2‐week follow‐up, he reported an improvement in his symptoms, which was accompanied by a marked radiological improvement. A repeat CT scan revealed a significant shrinkage of the right lower lobe consolidation to 22 × 16 mm, along with the complete resolution of the bilateral patchy opacities. The whole clinical course is presented in Figure 6 for a clearer view.

**Figure 6:**
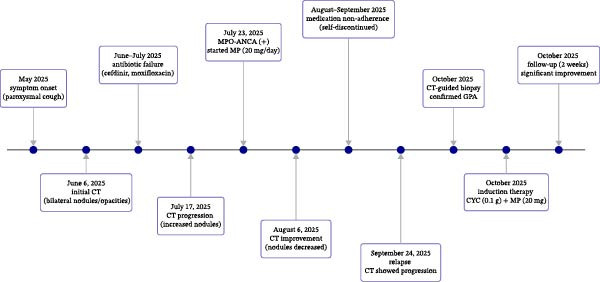
Clinical course of GPA patient (2025).

## 2. Literature Review

GPA, historically termed Wegener’s granulomatosis (WG), is a rare systemic vasculitis predominantly affecting small vessels. The disorder is characterized by necrotizing granulomatous inflammation and pauci‐immune complex deposition, most frequently involving the upper respiratory tract, lungs, and kidneys. While the classic pulmonary–renal syndrome represents a hallmark presentation, GPA is classified among the antineutrophil cytoplasmic antibody (ANCA)–associated vasculitides (AAV) [1]. Epidemiological studies indicate a prevalence of ~96.8 cases per million, with an annual incidence of 9.0 per million showing a progressive increasing trend. The disease shows a predilection for adults, with peak incidence between 50 and 70 years, a slight male predominance, and a notable prevalence in Asian populations [2, 3].

The precise etiology of GPA remains incompletely understood, involving a complex interaction between genetic susceptibility and microbiological factors. Immunologically, GPA involves dysregulation of both innate and adaptive immunity, B‐cell dysfunction, and the production of ANCA, particularly those targeting proteinase 3 (PR3), which are considered pivotal drivers of inflammation [4]. Environmental triggers, such as infections, can provoke an aberrant immune response dominated by Th1/Th17 cytokines (e.g., IL‐17, TNF, and IFN‐γ). This inflammatory milieu, in concert with ANCA, leads to neutrophil activation, enhanced endothelial adhesion, and degranulation, culminating in vascular damage and granuloma formation [5]. Genetic links include α‐1–antitrypsin deficiency and PR3 gene abnormalities, while *Staphylococcus aureus* colonization and certain viral infections are suspected initiating factors. The formation of granulomas initiates with neutrophilic microabscesses leading to vascular occlusion. Unlike the well‐structured granulomas in sarcoidosis, GPA lesions exhibit disorganized architecture composed of multinucleated giant cells surrounded by plasma cells, lymphocytes, and dendritic cells, which mediate tissue necrosis and structural deformity [4, 5].

GPA presents with a broad spectrum of clinical features, often beginning with nonspecific systemic symptoms like fever and fatigue. As it evolves, multiorgan involvement emerges, a pattern that can mimic other conditions. The management is challenged by its relapsing nature, where subsequent flares may differ from the initial presentation. The most frequently affected sites are the otolaryngologic, upper respiratory, and pulmonary systems. More than 70% of patients first present with upper respiratory tract involvement, while a minority (5%–10%) experience a localized form confined to the ocular or otolaryngologic tracts. The oral cavity is a frequent initial site, with 6%–13% of patients developing manifestations like “strawberry gingivitis”—diffuse, granular gingival swelling that is unresponsive to conventional periodontal therapy. Other oral features include persistent ulcers, oronasal fistulae, mucosal nodules, and alveolar bone necrosis [6, 7]. Although this presentation may mimic periodontitis, it remains unresponsive to conventional periodontal therapy. Beyond gingival involvement, GPA can induce persistent oral ulcers with predilection for the palate and tongue, frequently demonstrating refractory healing. Additional oral manifestations include oronasal fistulae, mucosal nodules, alveolar bone necrosis, tooth mobility/loss, and labial swelling [8].

Otolaryngological manifestations occur in 80%–90% of GPA patients [9]. Auditory issues primarily present as otitis media and hearing loss, while the auricle may exhibit inflammatory signs. Nasal pathology includes inflammatory crusting, mucosal erythema, and a destructive process resulting in septal perforation or saddle nose deformity [10]. In our case, the patient’s otolaryngological involvement was detected exclusively through targeted diagnostic investigations without presenting corresponding clinical symptoms, a scenario that can easily lead to oversight by clinicians.

Pulmonary manifestations occur in 70%–80% of cases, commonly as cough, dyspnea, and hemoptysis [11]. Renal involvement develops in 50%–80% of cases, often as rapidly progressive crescentic glomerulonephritis, which frequently leads to chronic kidney disease [12]. Ocular manifestations affect over half of patients, predominantly as scleritis and conjunctivitis, which may advance to blindness [13]. Cutaneous involvement, seen in 30%–60% of cases, presents as palpable purpura, papulonodular lesions, or pyoderma gangrenosum–like ulcers [14]. Additionally, GPA can affect the gastrointestinal, nervous, and musculoskeletal systems [15].

Comprehensive laboratory investigation should include complete blood count, renal function tests, urinalysis, and inflammatory markers like ESR and CRP. Anemia and elevated leukocyte counts often reflect systemic inflammation, while renal involvement manifests as hematuria and proteinuria. During active phases, hypergammaglobulinemia or increased serum IgG4 may be observed. ANCA represents the most crucial diagnostic marker for GPA. The 2022 European Alliance of Associations for Rheumatology (EULAR) guidelines recommend high‐quality antigen‐specific assays for PR3‐ANCA and MPO‐ANCA as the primary methodology. c‐ANCA targeting PR3 achieves 95% specificity for GPA, with over 70% of patients testing positive. However, a minority show MPO‐ANCA reactivity [1].

Imaging evaluation identifies organ involvement and guides biopsy. Chest CT is superior to radiography in differentiating GPA from infections and assessing pulmonary hemorrhage [16]. Characteristic manifestations include nodules and infiltrates in mid‐lung zones, frequently with cavitary lesions, as seen in this case where multiple nodules showed resolution following therapy [17, 18]. This case featured multiple pulmonary nodules that showed significant resolution following corticosteroid therapy. Bronchoalveolar lavage provides valuable diagnostic information regarding pulmonary infiltrates, particularly in detecting alveolar hemorrhage or eosinophilic alveolitis, alongside facilitating microbiological assessment of the lower respiratory tract. Sinus CT in patients with otolaryngological involvement typically reveals mucosal thickening, sinusitis, and mastoiditis, with advanced cases demonstrating bone destruction [19]. Magnetic resonance imaging (MRI) effectively detects central nervous system lesions, pachymeningitis, retro‐orbital pathology, and subglottic inflammation [20], while positron emission tomography‐computed tomography (PET‐CT) can identify occult sites of disease activity, concurrent malignancies, and chronic infections [21].

A tissue biopsy is indispensable for confirming GPA [22]. Due to the commonality of renal disease, a renal biopsy is most often employed, providing critical information for the initial diagnosis and relapse evaluation in AAV. It achieves diagnostic confirmation in ~91.5% of cases and simultaneously offers valuable prognostic insights. In the pulmonary tissue, the classic histopathological picture shows granulomas with palisading histiocytes and giant cells encircling a central necrotic core, which causes coagulative or liquefactive necrosis of the lung architecture. The vascular inflammation in these areas typically involves both arteries and veins and demonstrates a mixed inflammatory infiltrate rich in neutrophils, eosinophils, and plasma cells, with occasional lymphocytes [22].

Currently, no universally standardized international diagnostic criteria exist for GPA, though serum ANCA positivity serves as a crucial serological marker for clinical suspicion. The 2022 classification criteria jointly developed by the American College of Rheumatology (ACR) and the EULAR (Table S1) have gained widespread adoption [1]. This integrated framework incorporates clinical manifestations, laboratory findings, imaging features, and histopathological evidence utilizing a weighted scoring system (with a total score ≥ 5 points indicating GPA classification, detailed in Supporting Information). The criteria demonstrate excellent diagnostic performance with 93% sensitivity and 94% specificity. Their principal advantages include the incorporation of ANCA serology and modern imaging modalities, providing broad applicability and effective differentiation between GPA and other vasculitides.

Treatment is divided into induction of remission and maintenance therapy [23]. The induction phase aims for rapid disease control to avert irreversible organ damage, while maintenance preserves long‐term control. Successful therapy culminates in sustained remission, ideally allowing for the cessation of immunosuppressants [24, 25].

Induction therapy for GPA combines glucocorticoids with an immunosuppressive agent, selected based on disease severity. In severe, organ‐threatening disease, guidelines recommend glucocorticoids plus rituximab (preferred in relapse) or cyclophosphamide (Grade A); IV methylprednisolone and plasma exchange may be added [23]. For nonorgan‐threatening diseases, glucocorticoids with rituximab are standard, with methotrexate or mycophenolate mofetil as potential substitutes. Avacopan can replace glucocorticoids alongside RTX/CYC. Notably, for patients with severe or rapidly worsening renal failure (serum creatinine > 4 mg/dL), a regimen combining both rituximab and cyclophosphamide is an option.

Upon achieving remission, patients with GPA transition to a maintenance therapy phase. This long‐term strategy typically combines low‐dose glucocorticoids with an immunosuppressive agent, with rituximab representing the first‐line choice. Alternatively, azathioprine or methotrexate may be used.

Immunosuppressive and biologic therapies have significantly enhanced survival in GPA, with current 5‐year rates ranging from 74% to 91%. Long‐term outcomes are influenced by two key determinants: the degree of permanent organ damage and the cumulative burden of immunosuppression. The presence of severe renal disease, in particular, is independently linked to a poorer prognosis and elevated mortality [26].

This case presents a classic diagnostic challenge of GPA manifesting with an atypical serological profile. Although GPA is traditionally associated with PR3‐ANCA, this patient demonstrated MPO‐ANCA positivity. A notable feature was the discrepancy between the two MPO‐ANCA tests: The first was positive (49.7 AU/mL), while the second returned negative. This conversion likely reflects the rapid effect of corticosteroid therapy initiated after admission, though laboratory variance cannot be entirely ruled out. Such fluctuations underscore the importance of early serological testing before the initiation of immunosuppressive treatment to avoid masking critical diagnostic markers.

Despite the serological ambiguity, the diagnosis was firmly established by integrating clinical manifestations with histopathological evidence, adhering to the 2022 ACR/EULAR classification criteria (Table S1). The patient exhibited a triad of clinical involvement: the upper respiratory tract (sinusitis), the otolaryngological system (bilateral hearing loss), and the pulmonary system (nodules). The radiological “migration” and progression of nodules despite broad‐spectrum antibiotics provided a strong clinical clue toward noninfectious granulomatous disease.

The definitive evidence was provided by the CT‐guided lung biopsy, which revealed granulomatous inflammation and, crucially, partial disruption of elastic fibers—a hallmark of necrotizing vasculitis. Furthermore, the patient’s clinical course offered a cautionary lesson on management; the rapid radiological relapse following self‐discontinuation of medication confirms the aggressive nature of GPA and the necessity of strict adherence to maintenance therapy. This case emphasizes that when clinical and pathological evidence strongly points toward GPA, a single negative or atypical ANCA result should not deter the diagnosis or the prompt initiation of intensive induction therapy.

## Author Contributions

Conceptualization and investigation: Yanghang Chen, Liuyang Gong, and Haihong Zheng. Methodology, software, validation, formal analysis, visualization, and supervision: Yanghang Chen. Resources, data curation, and writing – original draft preparation: Yanghang Chen and Liuyang Gong. Writing – review and editing: Yanghang Chen and Haihong Zheng. Project administration and funding acquisition: Yanghang Chen.

## Funding

This research did not receive any specific grant from funding agencies in the public, commercial, or not‐for‐profit sectors.

## Disclosure

All authors have read and agreed to the published version of the manuscript.

## Ethics Statement

This retrospective study involving human participants was approved by the Ethics Committee of Taizhou Hospital. The need for informed consent was waived by the ethics committee due to the retrospective nature of the study and the use of anonymized data. All procedures performed in this study were in accordance with the ethical standards of the Declaration of Helsinki.

## Conflicts of Interest

The authors declare no conflicts of interest.

## Supporting Information

Additional supporting information can be found online in the Supporting Information section.

## Supporting information


**Supporting Information** This supporting information provides additional information and data related to the study presented in the main manuscript. It includes Table S1: The 2022 ACR/EULAR Classification Criteria for Granulomatosis with Polyangiitis (GPA), detailing the weighted scoring system across clinical, laboratory, imaging, and biopsy domains. This supporting information is essential for providing further evidence for the clinical diagnosis of the presented case. It supports the conclusions by demonstrating how the patient’s clinical manifestations (such as hearing loss and sinusitis) and diagnostic findings (such as positive ANCA and pulmonary nodules) fulfill the standardized international criteria for GPA classification.

## Data Availability

Data sharing is not applicable to this article as it is a case study. The case data and key information supporting the findings are fully presented within the manuscript and the provided supporting information. To protect patient privacy, the raw clinical data are not suitable for public sharing but can be made available from the corresponding author upon reasonable request.
