# Supplementary material for: Granulomatosis With Polyangiitis Presenting as Pulmonary Nodules: A Case Study and Literature Review
Source: Case Reports Immunol. 2026 Jul 27;2026:6977376. doi: 10.1155/crii/6977376 (PMC13402936; doi:10.1155/crii/6977376)
Supplement: Supplementary file 1 — Supporting Information This supporting information provides additional information and data related to the study presented in the main manuscript. It includes Table S1: The 2022 ACR/EULAR Classification Criteria for Granulomatosis with Polyangiitis (GPA), detailing the weighted scoring system across clinical, laboratory, imaging, and biopsy domains. This supporting information is essential for providing further evidence for the clinical diagnosis of the presented case. It supports the conclusions by demonstrating how the patient’s clinical manifestations (such as hearing loss and sinusitis) and diagnostic findings (such as positive ANCA and pulmonary nodules) fulfill the standardized international criteria for GPA classification. [file CRII-2026-6977376-s001.docx]

**Supplementary materials**

****Supplementary Table S1:****

****2022 ACR/EULAR Classification Criteria for Granulomatosis with Polyangiitis (GPA)****

| Domain / Item | Criteria | Score |
| --- | --- | --- |
| ****Clinical Criteria**** |  |  |
|  | Nasal bleeding, ulcers, crusting, congestion, or obstruction, OR nasal septal defect/perforation | 3 |
|  | Cartilaginous involvement (inflammation of ear/nasal cartilage, hoarseness/strider, bronchial involvement, OR saddle nose deformity) | 2 |
|  | Conductive or sensorineural hearing impairment | 1 |
| ****Laboratory, Imaging, and Biopsy Criteria**** |  |  |
|  | Positive cytoplasmic ANCA (c-ANCA) OR anti-proteinase 3 (PR3) antibodies | 5 |
|  | Chest imaging showing: pulmonary nodules, mass, or cavities | 2 |
|  | Biopsy showing granulomas, extravascular granulomatous inflammation, OR giant cells | 2 |
|  | Imaging showing: nasal/sinus inflammation, opacification, or fluid, OR mastoiditis | 1 |
|  | Biopsy showing pauci-immune glomerulonephritis | 1 |
|  | Positive perinuclear ANCA (p-ANCA) OR anti-myeloperoxidase (MPO) antibodies | -1 |
|  | Serum eosinophil count ≥1×10⁹/L | -4 |
|  | ****Total Score**** |  |
|  | A total score of ≥5 is classified as Granulomatosis with Polyangiitis. |  |
